# Initial responsiveness to darbepoetin alfa and its contributing factors in non-dialysis chronic kidney disease patients in Japan

**DOI:** 10.1007/s10157-020-01969-7

**Published:** 2020-09-19

**Authors:** Terumasa Hayashi, Hideki Kato, Kenichiro Tanabe, Masaomi Nangaku, Hideki Hirakata, Takashi Wada, Hiroshi Sato, Yasushi Yamazaki, Takao Masaki, Tatsuo Kagimura, Hiroyasu Yamamoto, Hiroki Hase, Masahiro Kamouchi, Enyu Imai, Kyoichi Mizuno, Manabu Iwasaki, Tadao Akizawa, Yoshiharu Tsubakihara, Shoichi Maruyama, Ichiei Narita

**Affiliations:** 1 Department of Kidney Disease and Hypertension, Osaka General Medical Center, Osaka, Japan; 2 Division of Nephrology and Endocrinology, The University of Tokyo Graduate School of Medicine, Tokyo, Japan; 3 Translational Research Center for Medical Innovation, Kobe, Japan; 4 Fukuoka Renal Clinic, Fukuoka, Japan; 5 Department of Nephrology and Laboratory Medicine, Faculty of Medicine, Institute of Medical, Pharmaceutical and Health Sciences, Kanazawa University, Kanazawa, Japan; 6 Division of Clinical Pharmacology and Therapeutics, Tohoku University Graduate School of Pharmaceutical Sciences and Faculty of Pharmaceutical Sciences, Sendai, Japan; 7 Department of Nephrology and Rheumatology, Kagawa Prefectural Central Hospital, Takamatsu, Japan; 8 Department of Nephrology, Hiroshima University Hospital, Hiroshima, Japan; 9 Division of Nephrology and Hypertension, Department of Internal Medicine, The Jikei University School of Medicine, Tokyo, Japan; 10 Division of Nephrology, Toho University Ohashi Medical Center, Tokyo, Japan; 11 Department of Health Care Administration and Management, Graduate School of Medical Sciences, Kyushu University, Fukuoka, Japan; 12 Nakayamadera Imai Clinic, Takarazuka, Japan; 13 Mitsukoshi Health and Welfare Foundation, Tokyo, Japan; 14 School of Data Science, Yokohama City University, Yokohama, Japan; 15 Division of Nephrology, Department of Medicine, Showa University School of Medicine, Tokyo, Japan; 16 Course of Safety Management in Health Care Sciences, Graduate School of Health Care Sciences, Jikei Institute, Osaka, Japan; 17 Department of Nephrology, Nagoya University Graduate School of Medicine, Nagoya, Japan; 18 Division of Clinical Nephrology and Rheumatology, Niigata University Graduate School of Medical and Dental Sciences, 757 Ichibancho Asahimachidori Chuo-ku, Niigata, 951-8510 Japan

**Keywords:** Erythropoiesis-stimulating agents, Hyporesponsiveness, Darbepoetin alfa, Chronic kidney disease, Pre-dialysis

## Abstract

**Background:**

Hyporesponsiveness to erythropoiesis-stimulating agents (ESAs) is associated with cardiovascular events and poor renal outcome in patients with chronic kidney disease (CKD). This study aimed to investigate the initial responsiveness to darbepoetin alfa (DA) and its contributing factors using the data from the BRIGHTEN.

**Methods:**

Of 1980 patients enrolled at 168 facilities, 1695 were included in this analysis [285 patients were excluded mainly due to lack of hemoglobin (Hb) values]. The initial ESA response index (iEResI) was defined as a ratio of Hb changes over 12 weeks after DA administration per weight-adjusted total DA dose and contributing factors to iEResI were analyzed.

**Results:**

The mean age was 70 ± 12 years (male 58.8%; diabetic nephropathy 27.6%). The median creatinine and mean Hb levels at DA initiation were 2.62 mg/dL and 9.8 g/dL, respectively. The most frequent number of DA administration during 12 weeks was 3 times (41.1%), followed by 4 (15.6%) times with a wide distribution of the total DA dose (15–900 μg). Remarkably, 225 patients (13.3%) did not respond to DA. Multivariate analysis showed that male gender, hypoglycemic agent use, iron supplementation, high eGFR, low Hb, low CRP, low NT-proBNP, and low urinary protein–creatinine ratio were independently associated with better initial response to DA (*P* =  < 0.0001, 0.0108, < 0.0001, 0.0476, < 0.0001, 0.0004, 0.0435, and 0.0009, respectively).

**Conclusions:**

Non-responder to DA accounted for 13.3% of patients with non-dialysis CKD. Iron supplementation, low CRP, low NT-proBNP, and less proteinuria were predictive and modifiable factors associated with better initial response to DA.

**Electronic supplementary material:**

The online version of this article (10.1007/s10157-020-01969-7) contains supplementary material, which is available to authorized users.

## Introduction

Anemia in patients with chronic kidney disease (CKD) is associated with poor renal, cardiovascular, and overall outcomes [1–5]. Observational studies and small randomized controlled trials (RCTs) showed that correction of anemia with erythropoiesis-stimulating agents (ESAs) was associated with beneficial outcomes in morbidity, mortality, and kidney function as well as quality of life in this patient population [6–11]. Furthermore, the beneficial effect of ESAs was greater with high hemoglobin (Hb) levels; however, the optimal target Hb level in ESA treatment remains controversial. A series of large RCTs comparing the effect of normalizing Hb level (> 13 g/dL) with conservative Hb level (10–11 g/dL) on mortality and cardiovascular disease (CVD) events was conducted over the past two decades and showed a consistent result of harm with normalizing Hb level [12–15]. The Correction of Hemoglobin and Outcomes in Renal Insufficiency (CHOIR) trial [14] and the Trial to Reduce Cardiovascular Events with Aranesp Therapy (TREAT) [15] conducted on pre-dialysis CKD patients showed that targeting higher Hb level compared with targeting lower Hb level increased the incidence of CVD events, especially stroke, and mortality. Furthermore, post hoc analyses of CHOIR and TREAT trials suggested that poor outcomes might not have resulted from achieving higher Hb level itself but resulted from toxicities associated with high-dose ESAs, patient-related factors promoting ESA hyporesponsiveness, or a combination of both [16, 17]. Thus, in the management of anemia for CKD patients, ESA hyporesponsiveness should be predicted and appropriate dose of ESA should be administered to patients with ESA hyporesponsiveness. However, clinically relevant definition of ESA hyporesponsiveness is not available at present because its definition should be based on the index associated with poor renal, cardiovascular, or overall outcome [18].

The “oBservational clinical Research In chronic kidney disease patients with renal anemia: renal proGnosis in patients with Hyporesponsive anemia To Erythropoiesis-stimulating agents, darbepoetiN alfa (BRIGHTEN)” is a multicenter prospective observational study conducted in a real-world clinical setting in Japan to explore the prevalence of hyporesponsiveness to darbepoetin alfa (DA) and to establish an appropriate index of hyporesponsiveness to DA associated with poor renal outcome and CVD events in non-dialysis CKD patients. Patient enrollment started in June 2014, and the observation period ended in September 2018. Therefore, this study aimed to investigate the initial responsiveness to DA and its contributing factors using the data from the BRIGHTEN.

## Materials and methods

### Study design

The study design and other details of the study protocol have been published elsewhere [19]. The protocol was approved by the main institutional review board (Nagoya University; No. 2014-0027) and then by each participating center. The research was conducted under the health insurance system of Japan and in accordance with the principles of the Declaration of Helsinki and Ethical Guidelines on Clinical Studies of the Ministry of Health, Labor, and Welfare of Japan. Written informed consent was provided by each participant. The research was designed, implemented, and overseen by the BRIGHTEN Executive Committee, together with representatives of Translational Research Center for Medical Innovation, Kobe, Japan, a third-party organization independent of the investigators’ institutions and responsible for data collection and analysis. The manuscript was prepared by one of the authors and subsequently revised and edited by all authors. The study was registered to ClinicalTrials.gov (NCT02136563) and UMIN-CTR (UMIN000013464).

### Study population

**Fig. 1:**
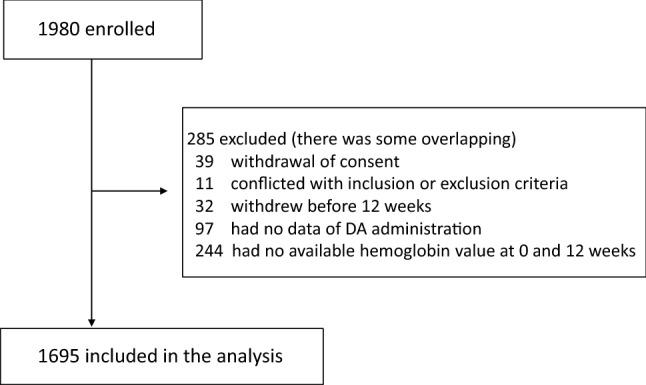
Screening of patient. *DA* darbepoetin alfa

Patients aged ≥ 20 years with estimated glomerular filtration rate (eGFR) of < 60 mL/min/1.73 m^2^ (calculated with the Japanese equation [20]) who presented renal anemia (Hb  < 11 g/dL) were enrolled from June 2014 to September 2016. Patients scheduled to initiate maintenance dialysis or undergo kidney transplantation until 24 weeks after registration; those with history of ESA treatment (administered with ESA temporarily and > 12 weeks before registration were eligible); and those with malignant tumors under treatment, hematologic diseases, or hemorrhagic diseases were excluded. Of 1980 patients enrolled in 168 facilities, 285 patients were excluded mainly due to the lack of Hb values at 0 and 12 weeks (84 ± 14 days). Finally, 1695 patients were included in the data analysis (Fig. 1). Patients were observed for 96 weeks since DA administration.

### DA administration

DA was administered within 8 weeks after the registration along its product information (30 μg every 2 weeks for the initial dose, subcutaneously or intravenously, and the dosage and duration should be adjusted thereafter to maintain Hb levels at ≥ 11 g/dL); however, the dose adjustment was actually entrusted to the physicians’ discretion in each facility as the BRIGHTEN was conducted in a real-world clinical setting.

### Data collection

Patient baseline characteristics (age, sex, etiology of CKD, smoking status, medical history, comorbidities, hypoglycemic agent use, renin–angiotensin system inhibitor use, iron supplementation, body mass index, and blood pressure) were collected at the study registration. The clear definition of diabetes and dyslipidemia was not adopted for the BRIGHTEN, while the diagnosis was entrusted to investigators’ judgement.

Complete blood count including Hb, serum creatinine, albumin, iron, ferritin, total iron-binding capacity (TIBC), hemoglobin A1c and urinary protein–creatinine ratio (PCR) were measured at each facility laboratory at the beginning of the study and on week 12. In addition, high-sensitivity C-reactive protein (CRP), folic acid, vitamin B12, aminoterminal pro-brain natriuretic peptide (NT-pro BNP), iron, ferritin, and TIBC were measured at the clinical laboratory company (SRL, Tokyo, Japan).

### Response index to DA

For the assessment of ESA hyporesponsiveness, the use of the following formulae (ESA resistance index; ERI [21]) were originally planned.

$$ {\text{ERI } - \text{ 1A}}\, = \,\frac{{{\text{Dose of DA at }}12{\text{ weeks }}\left( {{\mu g}} \right)}}{{{\text{Concentration of Hb }}\left( {{\text{g}}/{\text{dL}}} \right){\text{ at }}12{\text{ weeks }} \times {\text{ body weight }}\left( {{\text{kg}}} \right)}}, $$

$$ {\text{ERI } - \text{ 1B}}\, = \,\frac{{{\text{Dose of DA at }}12{\text{ weeks }}\left( {{\mu g}} \right)}}{{{\text{Concentration of Hb }}\left( {{\text{g}}/{\text{dL}}} \right){\text{ at }}12{\text{ weeks}}}}, $$

$$ {\text{ERI } - \text{ 2A}}\, = \,\frac{{{\text{Total dose of DA during }}12{\text{ weeks }}\left( {{\mu g}} \right)}}{{\Delta {\text{Hb }}_{0 - 12}\left( {{\text{g}}/{\text{dL}}} \right){ } \times {\text{ body weight }}\left( {{\text{kg}}} \right)}}, $$

$$ {\text{ERI } - \text{ 2B}}\, = \,\frac{{{\text{Total dose of DA during }}12{\text{ weeks }}\left( {{\mu g}} \right)}}{{\Delta {\text{Hb }}_{0 - 12}\left( {{\text{g}}/{\text{dL}}} \right)}}, $$

where ΔHb _0–12_ (g/dL) = Hb (g/dL) at 12 weeks—Hb (g/dL) before DA administration.

However, some patients showed decreased or no changes in Hb level (ΔHb _0–12_ < 0 or = 0) during 12 weeks after DA administration; thus, these aforementioned formulae were not used in the data analysis. Instead, initial ESA response index (iEResI) was defined as a reciprocal of the ERI-2A.

$$ {\text{iEResI}}\, = \,\frac{{\Delta {\text{Hb }}_{0 - 12}\left( {{\text{g}}/{\text{dL}}} \right) \times {\text{ body weight }}\left( {{\text{kg}}} \right)}}{{{\text{Total dose of DA during }}12{\text{ weeks }}\left( {{\mu g}} \right)}}. $$

### Statistical analysis

Baseline characteristics are reported as means ± standard deviation (SD), median [interquartile range (IQR)], or number (percentage). The Wilcoxon rank sum test was used to compare two groups. As regards the predictors of initial response to DA, the association of iEResI with the number of DA administration and total doses of DA was firstly investigated. Furthermore, contributing factors to iEResI were analyzed using the general linear model adjusted by gender as a factor. We included all baseline demographic and clinical variables into univariate analysis; then variables significantly associated with iEResI (*P* < 0.05) were incorporated into multivariate analysis. All analyses were performed using SAS version 9.4 (SAS Institute, Cary, NC, USA), and *P* values of < 0.05 were considered significant.

## Results

The mean patient age was 70 ± 12 years (male 58.8%). Diabetic nephropathy was the leading cause of CKD (27.6%) followed by nephrosclerosis (23.5%) and chronic glomerulonephritis (22.5%). The prevalence of coronary artery disease, heart failure, stroke, and peripheral artery disease was 15. 8%, 7.0%, 11.7%, and 11.0%, respectively. Creatinine and Hb levels at DA initiation were 2.62 (1.89, 3.61) mg/dL and 9.8 ± 0.9 g/dL, respectively (Table1). The prevalence of patients with serum ferritin level < 50 μg and TSAT < 20% was 27.2% and 22.4%, respectively (Supplementary Fig. 1).

**Table 1:** Baseline characteristics

	Number of patients	Prevalence, mean ± SD, or median (IQR)
Age years	1695	70 ± 12
Male gender *n* (%)	–	997 (58.8)
Etiology of CKD		
Diabetic nephropathy *n* (%)	–	467 (27.6)
Chronic glomerulonephritis *n* (%)	–	381 (22.5)
Nephrosclerosis *n* (%)	–	398 (23.5)
Polycystic kidney disease *n* (%)	–	95 (5.6)
Other *n* (%)	–	354 (20.9)
Smoking status		
Current *n* (%)	–	183 (10.8)
Ever *n* (%)	–	617 (36.4)
Diabetes *n* (%)	–	722 (42.6)
Malignancy (past history) *n* (%)	–	208 (12.3)
Cardiovascular disease		
Coronary artery disease *n* (%)	–	268 (15.8)
Heart failure *n* (%)	–	118 (7.0)
Stroke *n* (%)	–	198 (11.7)
Peripheral artery disease *n* (%)	–	187 (11.0)
RAS inhibitor use		
Angiotensin II receptor blocker *n* (%)	–	972 (57.3)
Angiotensin converting enzyme inhibitor *n* (%)	–	179 (10.6)
Hypoglycemic agent use		
Dipeptidyl peptidase-4 inhibitor *n* (%)	–	362 (21.4)
Insulin *n* (%)	–	193 (11.4)
Iron supplementation *n* (%)	–	252 (14.9)
Body mass index (kg/m^2^)	1552	23.2 ± 4.0
Systolic arterial pressure (mmHg)	1603	134.3 ± 19.0
Diastolic arterial pressure (mmHg)	1601	71.3 ± 12.3
Creatinine (mg/dl)	1695	2.62 (1.89, 3.61)
Estimated glomerular filtration rate (ml/min/1.73m2)	1695	18.0 (12.9, 25.3)
Hemoglobin (g/dl)	1695	9.8 ± 0.9
Albumin (g/dl)	1652	3.7 ± 0.5
Ferritin (ng/ml)	1644	96.4 (46.4, 175.0)
Transferrin saturation (%)	1645	26.2 (20.6, 31.9)
High sensitive C-reactive protein (ng/dl)	1649	575 (219, 1810)
Folic acid (ng/ml)	1604	7.2 (5.5, 9.8)
Vitamin B12 (pg/ml)	1578	354 (258, 498)
NT-proBNP* (pg/ml)	1648	516.0 (238.5, 1160.0)
HbA1c (%)	1012	6.1 ± 0.9
Urinary protein-creatinine ratio (g/gCr)	1526	1.4 (0.5, 3.1)

*SD* standard deviation, *IQR* interquartile range, *CKD* chronic kidney disease, *RAS* renin-angiotensin system, *NT-proBNP* aminoterminal pro-brain natriuretic peptide

**Fig. 2:**
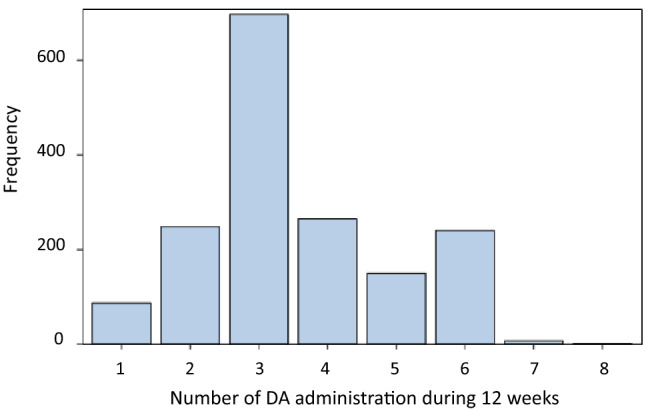
Number of DA administration during 12 weeks after DA initiation. *DA* darbepoetin alfa

**Fig. 3:**
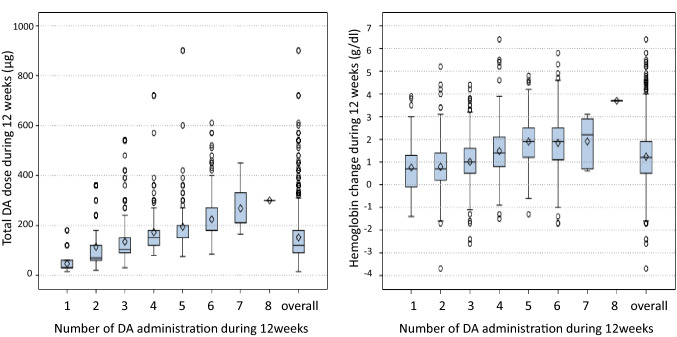
Total DA dose and hemoglobin change during 12 weeks after DA initiation stratified by the number of DA administration. *DA* darbepoetin alfa

The most frequent number of DA administration during 12 weeks was 3 times (*n* = 697, 41.1%), followed by 4 (*n* = 265, 15.6%) and 2 (*n* = 248, 14.6%) times with a wide distribution of the total DA dose ranging from 15 μg to 900 μg (Figs. 2, 3). The total DA dose and hemoglobin levels increased in relation to the number of DA administration for 12 weeks (Fig. 3).

**Fig. 4:**
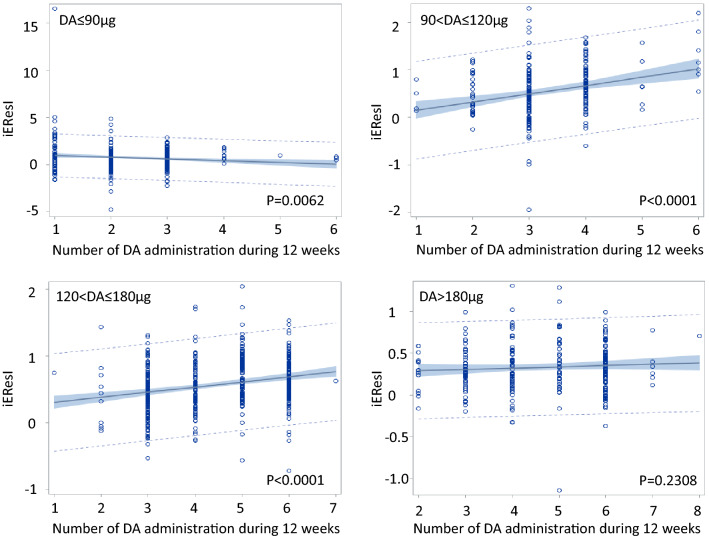
Association between iEResI and the number of DA administration stratified by the total DA dose until 12 weeks. iEResI increased with the number of DA administration, especially in the total DA dose between 90 μg and 180 μg; whereas, it was not associated with the total DA dose and even lower in the total DA dose of  > 180 μg. *DA* Darbepoetin alfa, *iEResI* initial response index to erythropoiesis-stimulating agents. Note: The scale of vertical axes in each graph is not unified.

iEResI increased with the number of DA administration, especially in the total DA dose between 90 μg and 180 μg; whereas, it was not associated with the total DA dose and even lower in the total DA dose of > 180 μg (Table 2 and Fig. 4).

**Table 2:** iEResI stratified by cumulative DA doses and number of DA administration

Cumulative DA dose	Number of DA administration	Number of patients	Mean	Standard deviation
≤ 90 μg	1	54	1.4120	2.6105
	2	135	0.5363	1.1022
	3	293	0.6012	0.6158
	4	10	1.0538	0.5824
	5	1	0.9949	–
	6	3	0.7087	0.1833
	Overall	496	0.6823	1.1659
> 90 μg and ≤ 120 μg	1	4	0.4073	0.3080
	2	31	0.4675	0.4046
	3	125	0.4212	0.5637
	4	80	0.6923	0.4602
	5	6	0.7412	0.5405
	6	7	1.2840	0.5611
	Overall	253	0.5438	0.5369
> 120 μg and ≤ 180 μg	1	1	0.7467	–
	2	10	0.3996	0.4987
	3	142	0.4237	0.3584
	4	95	0.5354	0.3770
	5	96	0.7171	0.3853
	6	130	0.6380	0.3328
	7	1	0.6204	–
	Overall	475	0.5645	0.3794
> 180 μg	1	0	–	–
	2	16	0.2714	0.2156
	3	48	0.3005	0.2492
	4	60	0.3238	0.3194
	5	40	0.3780	0.3925
	6	99	0.3343	0.2603
	7	6	0.3473	0.2317
	8	1	0.7055	–
	Overall	270	0.3304	0.2917

Notably, 225 patients (13.3%) had no response or even had reduced Hb level at week 12 (DA hyporesponsiveness group). A similar pattern of histograms of total DA dose during 12 weeks in two groups with and without DA hyporesponsiveness was observed; however, there was a statistically significant difference in the median cumulative DA dose between two groups with [100 (60, 180) μg] and without DA hyporesponsiveness [140 (90, 180) μg] (*P* < 0.0001) (Supplementary Fig. 2).

Multivariate analysis showed that male gender, hypoglycemic agent use, iron supplementation, not serum ferritin or TSAT level, and high eGFR were independently and significantly associated with better initial response to DA (*P* =  < 0.0001, 0.0108, < 0.0001, and 0.0476, respectively); whereas, high Hb, high CRP, high NT-proBNP, and high urinary PCR levels were significantly associated with poor initial response to DA (*P* =  < 0.0001, 0.0004, 0.0435, and 0.0009, respectively) (Table 3).

**Table 3:** Predictors associated with iEResI

	Univariate^a^		Multivariate^b^	
Variables	β coefficient	*P* value	β coefficient	*P* value
Age	− 0.003	0.1187		
Male gender (reference, female)	0.165	< 0.0001	0.234	< 0.0001
Etiology of CKD		0.9418		
Diabetic nephropathy	0.030			
Chronic glomerulonephritis	0.051			
Nephrosclerosis	0.015			
Polycystic kidney disease	− 0.030			
Smoking status, never (reference, current)	− 0.107	0.2969		
Diabetes, yes (reference, no)	0.082	0.0396		
Past history of malignancy, yes (reference, no)	− 0.097	0.1032		
Cardiovascular disease (reference, no)				
Coronary artery disease, yes	0.043	0.4191		
Heart failure, yes	− 0.007	0.9228		
Stroke, yes	− 0.003	0.9543		
Peripheral artery disease, yes	0.019	0.7633		
RAS inhibitor use, yes (reference, no)	− 0.016	0.7340		
Hypoglycemic agent use, yes (reference, no)	0.085	0.0415	0.112	0.0108
Iron supplementation, yes (reference, no)	0.223	< 0.0001	0.240	< 0.0001
Body mass index	0.003	0.4960		
Systolic arterial pressure	− 0.001	0.2430		
Diastolic arterial pressure	0.001	0.4385		
Log (Estimated glomerular filtration rate)	0.133	0.0014	0.095	0.0476
Hemoglobin	− 0.111	< 0.0001	− 0.123	< 0.0001
Albumin	− 0.017	0.6501		
Log (Ferritin)	0.014	0.5018		
Log (Transferrin saturation)	− 0.090	0.0703		
Log (C-reactive protein)	− 0.031	0.0198	− 0.048	0.0004
Log (Folic acid)	0.005	0.9112		
Log (Vitamin B12)	-0.008	0.8511		
Log (NT-proBNP)	− 0.039	0.0189	− 0.039	0.0435
HbA1c	0.046	0.1418		
Log (Urinary protein–creatinine ratio)	− 0.051	0.0005	− 0.053	0.0009

*CKD* chronic kidney disease, *RAS* renin-angiotensin system, *NT-proBNP* aminoterminal pro-brain natriuretic peptide

^a^Adjusted by gender as a factor

^b^Adjusted for variables significantly associated with iEResI in univariate analysis

## Discussion

ESA hyporesponsiveness is a strong predictor of mortality and CVD events as well as poor renal survival in patients with CKD [16, 17, 21–25]. Although the definition of ESA hyporesponsiveness has been suggested in previous clinical guidelines for the treatment of anemia in patients with CKD, they were defined arbitrarily and were not associated with any outcomes [26, 27]. Only the Kidney Disease: Improving Global Outcomes (KDIGO) anemia guideline published in 2012 defined ESA hyporesponsiveness as “patients who have no increased Hb concentration from baseline after the first month of ESA treatment on appropriate weight-based dose [28]. This definition was based on the secondary analysis of the TREAT study, which revealed that diabetic pre-dialysis CKD patients with initial hyporesponsiveness to DA had poor cardiovascular outcome [17]. However, in the TREAT study, nearly half of study subjects had CVD and the DA dose used was quite different from those used in our clinical practice setting in Japan [15, 25]. Therefore, extrapolating this definition into clinical practice in Japan seems difficult. In this context, an evidence-based index of ESA hyporesponsiveness and clinical guidelines for anemia management in CKD patients with ESA hyporesponsiveness should be established in Japan.

In the current analysis, 225 patients (13.3%) showed no increase or even had decreased Hb level at 12 weeks after DA administration. This prevalence was similar to those reported in previous studies regardless of different definitions of ESA hyporesponsiveness adopted [29, 30]. Furthermore, the new index of initial responsiveness to DA (iEResI) was defined. Male gender, hypoglycemic agent use, iron supplementation, and high eGFR were positively associated with iEResI; whereas, high Hb, high CRP, high NT-proBNP levels, and high urinary PCR were negatively associated with iEResI. Previous studies have already shown that iron deficiency, decreased renal function, and inflammation were strong predictors for ESA hyporesponsiveness; however, reports regarding the association between gender difference or hypoglycemic agent use (proxy for diabetes) and ESA hyporesponsiveness are conflicting [23, 24, 29, 31]. In our study, iron supplementation, not serum ferritin or TSAT level, was associated with iEResI on the univariate and multivariate analysis. As shown in Supplementary Fig. 1, the prevalence of study subjects with iron deficiency (e.g., serum ferritin < 50 μg and TSAT < 20%) seems to be lower than that previously reported in dialysis-dependent CKD patients [32], which might make difficult to show significant and apparent relationship between these parameters of iron status and iEResI. The association between male gender and better initial responsiveness to DA may be a mathematical artifact. Namely, DA is administered through prefilled syringe at a dose of 15, 30, 40, 60, 120, or 180 μg for pre-dialysis CKD patients in Japan, not according to the body weight-adjusted dose for each patient. As the iEResI adopted in this study had a figure of “body weight” in the numerator, the iEResI value may be relatively higher in men than in women. Some studies reported that diabetes or insulin resistance was associated with ESA hyporesponsiveness in pre-dialysis CKD patients [29, 33]; whereas, others did not show any association between diabetes and ESA hyporesponsiveness in dialysis patients [23, 31]. In this study, patients with hypoglycemic agents use had higher prevalence of dyslipidemia than those without hypoglycemic agent use (67.6% vs. 49.7%; *P* < 0.0001). Statin use has also been reported to improve ESA responsiveness in dialysis and pre-dialysis CKD patients due to its anti-inflammatory effect [34, 35]. Although the prevalence of statin use was not obtained in this study, such a confounding factor may affect this association. Our results also showed that high urinary PCR was associated with ESA hyporesponsiveness, which is consistent with a previous report [24]. Although the exact mechanism by which proteinuria could affect the ESA responsiveness has not been clarified, proteinuria is a proxy for inflammation or histological damage of the kidney and for progressive kidney function decline which could affect endogenous erythropoietin production or response to ESA. Of interest, history of CVD was not associated with the iEResI in our study; whereas, NT-proBNP was significantly associated with the iEResI. As the aforementioned study showed that patients with history of heart failure not of coronary artery disease showed ESA hyporesponsiveness [23], history of CVD which has been usually used for adjustment of confounders in previous studies has a crucial week point, which means that the history of CVD can not represent the severity of CVD. On the other hand, BNP or NT-proBNP can represent the severity of CVD. Patients with severe CVD usually have more serious inflammation, or take more medication, which could be associated with anemia or poor response to ESA, compared to those with less severe CVD. Thus, we think that it is quite reasonable that NT-proBNP, not the history of CVD, was associated with the responsiveness to DA. Low Hb level before ESA treatment was reported as a predictor of ESA hyporesponsiveness [23, 29] because it means the presence of comorbidity or inflammation that could be directly associated with ESA hyporesponsiveness. Our study result showing the opposite association may be interpreted as patients with higher Hb level before DA initiation may be less dependent on erythropoietin. Age, malnutrition, and RAS inhibitor were also reported as predictive factors of ESA hyporesponsiveness [29, 36]; however, this study did not show any association between these factors and iEResI, although precise nutritional assessment other than serum albumin level was not performed for the BRIGHTEN.

Although the BRIGHTEN is nationwide prospective study that can closely monitor patients, several limitations should be considered. This study aimed to examine the initial responsiveness to DA in principally ESA-naïve patients. As ESA responsiveness is likely to change over time, 12 weeks may be relatively short to evaluate ESA responsiveness. As it is an observational study conducted in a real-world clinical setting, the dose and frequency of DA administration were not unified because they were determined based on the physician’s discretion and patients’ preference, which may affect the iEResI. Furthermore, there were many missing data about iron supplementation (type of agents, dose, and route of administration), so we could not analyze the relationship between the dose of iron supplementation and responsiveness to DA. Factors associated with ESA hyporesponsiveness could highly depend on patient characteristics studied, comorbid disease, and treatment for anemia itself (e.g., ESA dose); thus, our results could not be extrapolated to CKD patients in Western countries and to those undergoing chronic dialysis treatment.

In conclusion, in the current analysis of the BRIGHTEN, 13.3% of patients showed no increase or even decreased Hb level at 12 weeks after DA administration. Furthermore, male gender, hypoglycemic agent use, iron supplementation, high eGFR, low Hb, low CRP, low NT-proBNP, and low proteinuria were independently and significantly associated with better initial response to DA. In future analyses, we are planning to investigate the association between the iEResI as well as other ERIs and patient outcomes.

## Electronic supplementary material

Below is the link to the electronic supplementary material.

Supplementary file1 (PPTX 92 kb)
