# Supplementary figures and images for: Initial responsiveness to darbepoetin alfa and its contributing factors in non-dialysis chronic kidney disease patients in Japan
Source: Clin Exp Nephrol. 2020 Sep 19;25(2):110–9. doi: 10.1007/s10157-020-01969-7 (PMC7880978; doi:10.1007/s10157-020-01969-7)

## Slide 1
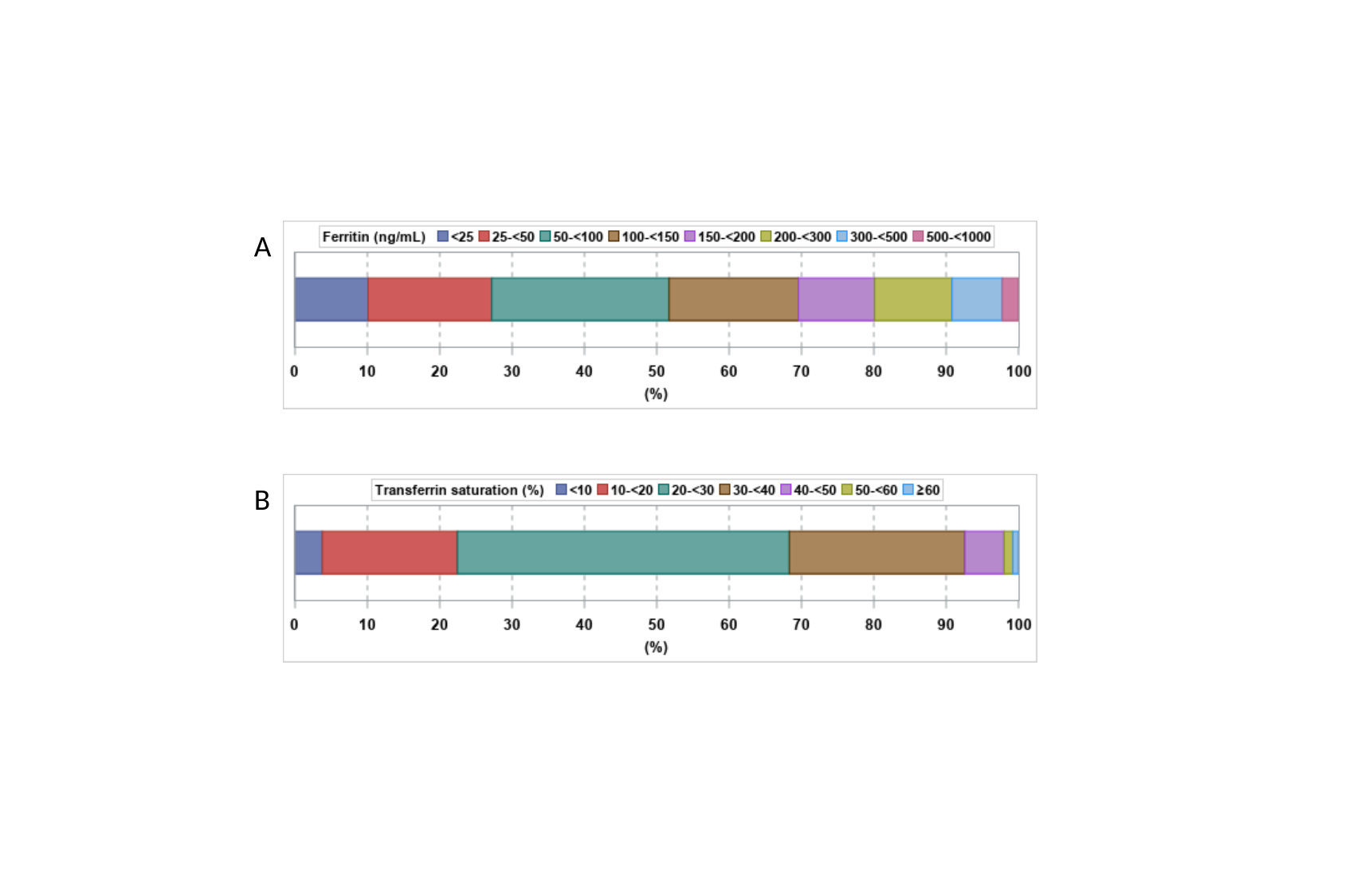

A
B

## Slide 2
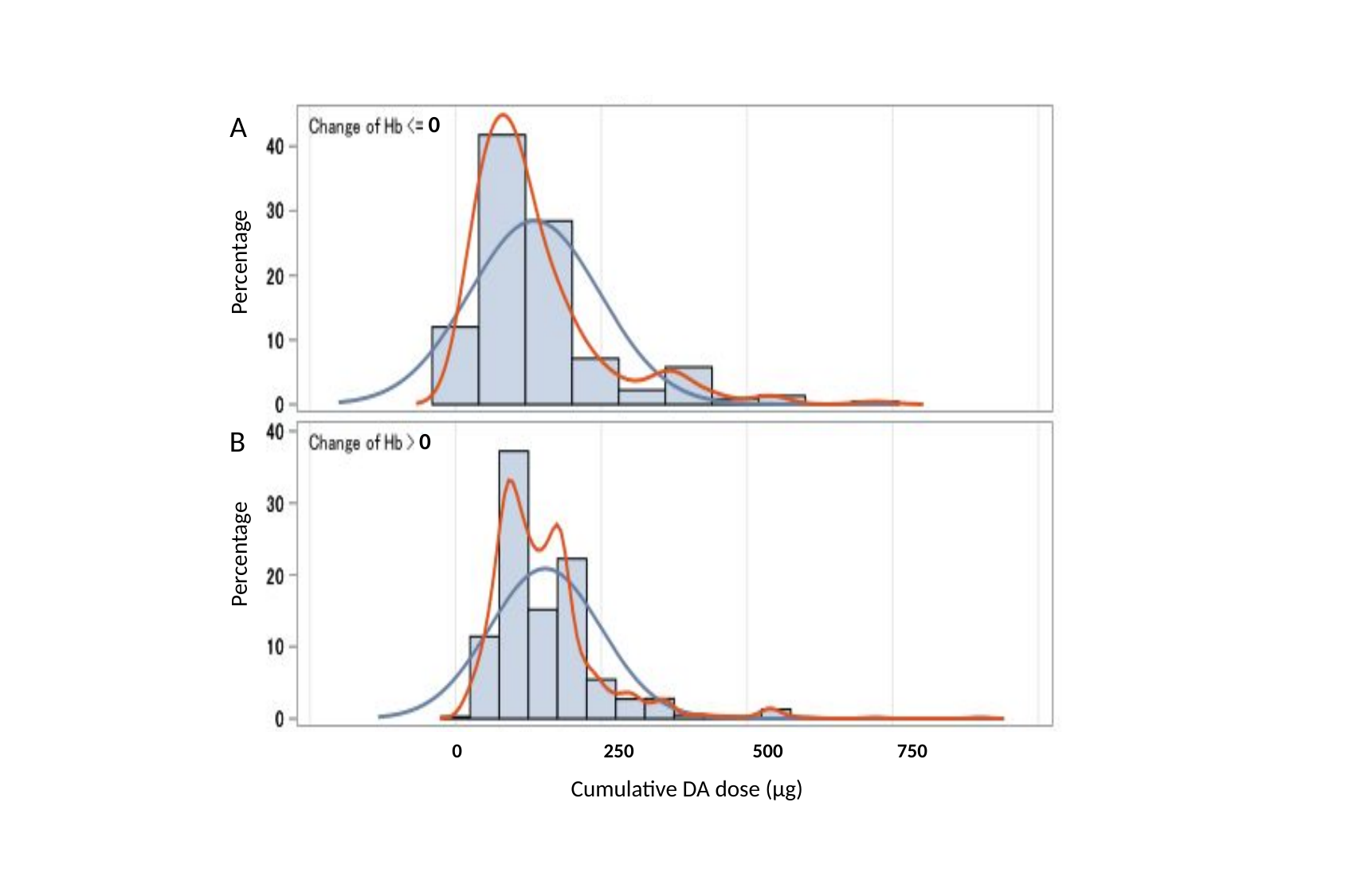

0
0 　　 250 500 750
Cumulative DA dose (μg)
Percentage
0
Percentage
A
B

Supplement: Supplementary file 1 — Supplementary file1 (PPTX 92 kb) [file 10157_2020_1969_MOESM1_ESM.pptx]
